# Chemical Effects on Breast Development, Function, and Cancer Risk: Existing Knowledge and New Opportunities

**DOI:** 10.1007/s40572-022-00376-2

**Published:** 2022-08-19

**Authors:** Jennifer E. Kay, Bethsaida Cardona, Ruthann A. Rudel, Laura N. Vandenberg, Ana M. Soto, Sofie Christiansen, Linda S. Birnbaum, Suzanne E. Fenton

**Affiliations:** 1 Silent Spring Institute, Newton, MA USA; 2 Department of Environmental Health Sciences, School of Public Health & Health Sciences, University of Massachusetts Amherst, Amherst, MA USA; 3 Tufts University School of Medicine, Boston, MA USA; 4 National Food Institute, Technical University of Denmark, Kongens Lyngby, Denmark; 5 Nicholas School of the Environment, Duke University, Durham, NC USA; 6 Mechanistic Toxicology Branch, Division of the National Toxicology Program, National Institutes of Environmental Health Sciences, National Institutes of Health, Durham, NC USA

**Keywords:** Endocrine disrupting chemical, Window of susceptibility, Weaning, Thelarche, Test guideline

## Abstract

Population studies show worrisome trends towards earlier breast development, difficulty in breastfeeding, and increasing rates of breast cancer in young women. Multiple epidemiological studies have linked these outcomes with chemical exposures, and experimental studies have shown that many of these chemicals generate similar effects in rodents, often by disrupting hormonal regulation. These endocrine-disrupting chemicals (EDCs) can alter the progression of mammary gland (MG) development, impair the ability to nourish offspring via lactation, increase mammary tissue density, and increase the propensity to develop cancer. However, current toxicological approaches to measuring the effects of chemical exposures on the MG are often inadequate to detect these effects, impairing our ability to identify exposures harmful to the breast and limiting opportunities for prevention. This paper describes key adverse outcomes for the MG, including impaired lactation, altered pubertal development, altered morphology (such as increased mammographic density), and cancer. It also summarizes evidence from humans and rodent models for exposures associated with these effects. We also review current toxicological practices for evaluating MG effects, highlight limitations of current methods, summarize debates related to how effects are interpreted in risk assessment, and make recommendations to strengthen assessment approaches. Increasing the rigor of MG assessment would improve our ability to identify chemicals of concern, regulate those chemicals based on their effects, and prevent exposures and associated adverse health effects.

## Background

The mammary gland (MG) is a hormone-sensitive organ that begins development in the womb and continues to grow and differentiate throughout an individual female’s lifetime, including puberty, pregnancy, and menopause [1, 2]. At each stage, hormonal signals mediate key biological changes that are necessary for subsequent phases of structural and functional development [2]. This continual dynamic development—with monthly cycles of proliferation and regression, full differentiation at the first full-term pregnancy, and more extensive regression after menopause—makes the organ vulnerable to disruption, with the potential for persistent effects for both a woman and her offspring [3]. Indeed, in recent decades, there have been increased reports of premature breast development [4–6], shortened duration of breastfeeding [7–9], and premenopausal breast cancer [10–13]. Despite these concerning trends, chemically induced alterations to MG development and function remain under-studied, while the need for research into the causes of these adverse outcomes is urgent to protect women and subsequent generations.

Hundreds of chemicals have been identified as endocrine disrupting chemicals (EDCs), and many of them have been linked to alterations in breast development, lactation, or cancer in both human cohorts and experimental studies in rodents [14–17]. However, most EDCs have not been evaluated for adverse mammary effects in regulatory evaluations. One important gap is lack of testing during sensitive phases of development, often termed windows of susceptibility (WoS) [3, 18–20]. It is likely that hundreds more endocrine-active mammary toxicants would be identified if mammary tissues were carefully evaluated in regulatory testing and if these studies included gestational and developmental exposures [21, 22].

While changing policies and practices have reduced exposures to some EDCs (such as dioxins and brominated flame retardants), others have become increasingly common or newly introduced (e.g., bisphenol A (BPA) analogs and per- and polyfluoroalkyl substances (PFAS)). Many EDCs and breast carcinogens are found in ubiquitous sources including consumer products, dust, food, and drinking water [14, 22, 23], exposing women and children to these chemicals throughout the life course, including during key WoS. Since many EDCs are pervasive in everyday life, individuals are exposed to them as mixtures, presenting risks for additive or even synergistic effects [24, 25]. As such, it is likely that current trends in MG pathologies, such as disruption in breast development, impaired lactation, and cancer, are driven, at least in part, by the cumulative impact of various environmental chemical exposures. The ability of EDCs to promote these pathologies is further suggested by evidence that women of color are disproportionately exposed to EDCs, and they have higher rates of alterations in pubertal timing, are less likely to breastfeed, and have worse breast cancer prognoses [10, 26–29]. Importantly, however, since EDCs and mixtures are unavoidable in daily life, it is difficult for epidemiological studies to pinpoint effects of any single chemical, making controlled experiments in rodents imperative to identify harmful exposures.

**Fig. 1:**
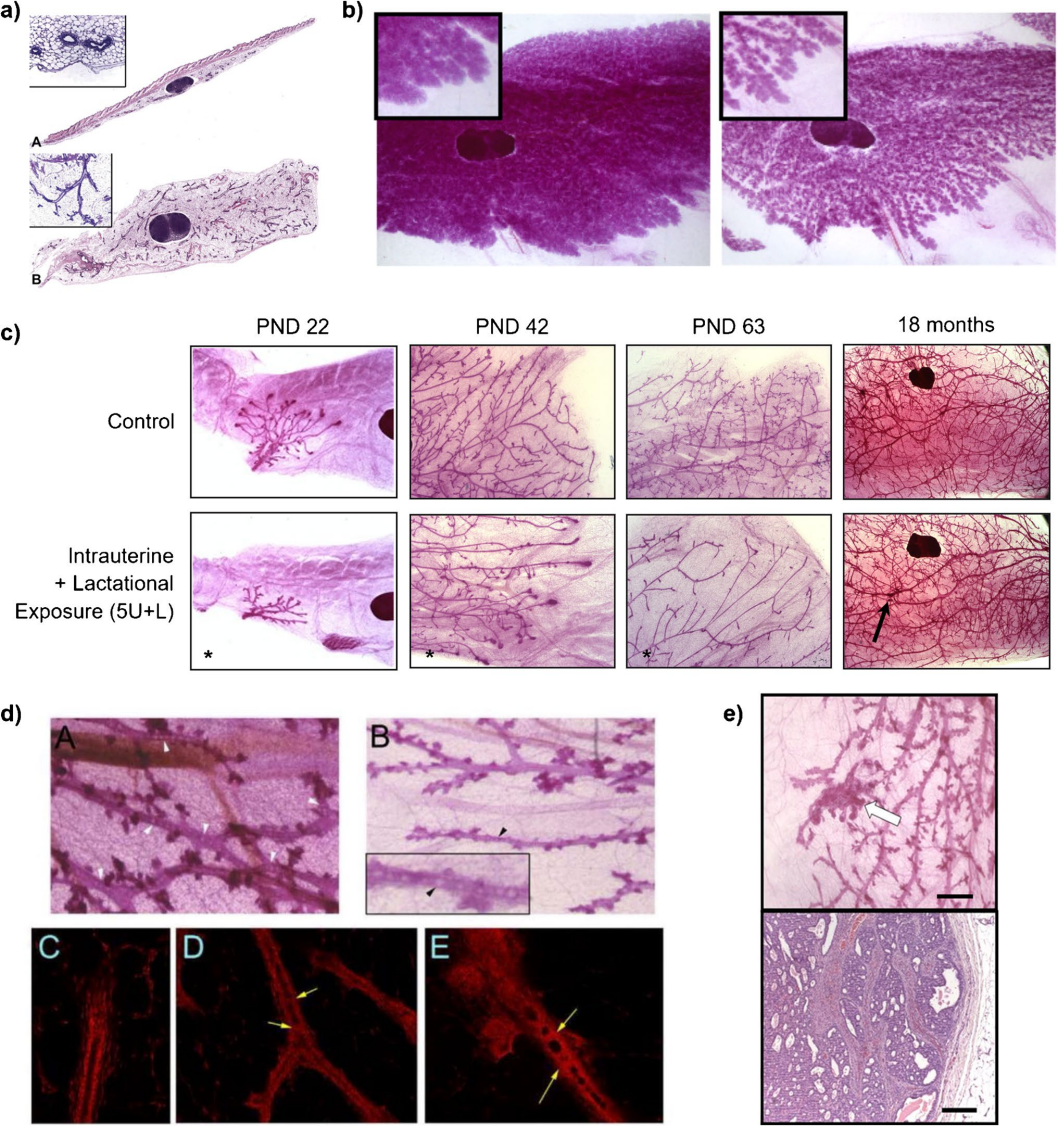
Mammary gland whole mounts provide detailed morphology and evidence of functional alterations. **a** Traditional histological sections of MG tissue. Classical transverse cross-section of the MG including skin (top; required by some test guidelines) shows very little visible MG epithelial cells for evaluation. Longitudinal section (bottom) of the MG presents larger area and more epithelial structures than transverse cross-section, but ductal tree structure may not be able to be evaluated. Figure adapted from Davis and Fenton, 2013 with permission [206]. **b** Impairment of lactation. MG whole mounts from mouse dams dosed with vehicle (left) or 200 μg BPS/kg/day (right) from gestation day 8 through lactation day 21. On lactation day 21, the vehicle-treated MG (left) remained full of an expansive network of lobuloalveolar units, whereas the BPS-exposed MG contained stunted and/or regressed alveolar buds. Magnification 6 × . (Unpublished, LN Vandenberg). **c** MG whole mounts from mice exposed to 5 mg/kg/day PFOA in utero and during lactation (lower panels) showed delays in ductal elongation, ductal branching, and terminal end bud formation relative to controls (upper panels). Developmental delays were apparent at puberty (PND22) and persisted into adulthood at PND63. At 18 months of age, epithelial density remained reduced in PFOA-exposed mice and lesions become apparent (arrow). Magnification 2.5 × for PND22, 5 × for PND42, 5 × for PND63, 1.6 × for 18 months. Figure adapted from White et al. 2009 with permission [69]. **d** Intraductal hyperplasia (beaded ducts) in MG whole mounts in female mice perinatally exposed to BPA (gestation day 8 to PND21). Unlike controls (upper left whole mount and lower left fluorescence; 9-month-old mice), the ductal lumens of BPA-treated mice (upper right whole mount (9-month) and lower middle (3-month) and right (9-month) fluorescence) contained cellular infiltrates, creating a “beaded” appearance. Upper panels magnification 32 × , lower panels magnification 200 × . Figure adapted from Vandenberg et al. 2008 with permission [227]. **e** Ductal carcinoma in situ. Top panel: Whole mount of a MG from a rat exposed to 2.5 μg BPA/kg/day from gestation day 9 until PND1, treated with a subcarcinogenic dose of MNU (20 mg/kg) at 50 days of age, and sacrificed 20 weeks after MNU injection. The arrow points to a lesion in a whole mount. No lesions were observed in the vehicle-treated animals. Scale bar = 1 mm. Bottom panel: H&E staining of the tumor removed from the lesion in the top panel. Scale bar = 200 μm. (Unpublished, AM Soto)

This review describes evidence showing that environmental chemicals contribute to four breast-relevant endpoints: (1) lactation, (2) morphological/structural changes as they relate to pubertal timing (thelarche), (3) breast density, and (4) breast cancer (as shown in Fig. 1). While a systematic literature search was outside the scope of this review, each section highlights several chemicals with evidence from epidemiological or experimental studies, particularly in rodents. We also discuss cross-cutting issues that are applicable to measuring these endpoints and assessing risks from chemical exposures that cause them. Topics reviewed are (1) gaps in test guidelines and practices used to evaluate MG effects in toxicology studies and approaches to address them; (2) the role of exposure timing, especially during WoS, on the induction of adverse MG outcomes; and (3) the classification of chemicals that alter MG developmental timing or tissue morphology as teratogens (i.e., chemicals that induce congenital malformations). We provide recommendations to strengthen toxicological and risk assessments.

## Lactation

In rodent models, there is increasing experimental evidence that environmental exposures, such as pesticides [30, 31], persistent organic pollutants [32–36], and some drugs [37], impair postnatal offspring weight gain and that those deficits may be related to the chemical’s effect on lactation. Further evidence suggests that exposures to these chemicals affect the MG by impairing proliferation and differentiation of mammary epithelial cells during development, altering involution of the gland, or disrupting the hormonal processes responsible for breast milk production (e.g., prolactin secretion and signaling and milk protein expression) [30, 32–35, 37–39]. Such chemically induced impairments in lactogenesis could partially explain why breastfeeding rates in women in high income countries are lower than recommended by health organizations [40, 41], with a majority of mothers citing insufficient milk supply as a potential reason for lack of appropriate weight gain in their infant, concern about the nutritional quality of their milk, and pain as reasons for early weaning [41–44]. Recent studies evaluating lactational parameters in the postpartum year have confirmed that a significant percentage of women abandon breastfeeding over concerns of insufficient milk production [45].

### Epidemiological Studies

Studies of environmental chemicals and lactation in women are limited, but several associations have emerged from epidemiological studies (Table 1). For instance, smoking has been found to reduce breast milk supply and quality [46, 47], which may be related to inhalation of chemical additives and combustion byproducts, many of which are EDCs [14]. In some populations, maternal smoking status during the postpartum period is associated with an increased likelihood of breastfeeding termination [48, 49], although these associations could be more related to lifestyle and social factors rather than effects of chemical exposures. While the causal pathway between these parameters is not established, a systematic review of 20 studies revealed that smoking is associated with lower milk lipid, protein, and caloric content; decreased antioxidant properties; and altered immune markers in breast milk [50], all of which can affect the health of the nursing offspring. These functional alterations suggest that, while lifestyle factors may play a role in nursing behavior, effects from chemical exposures likely also contribute to some mothers’ decisions to terminate breastfeeding early by altering milk quality or production.

**Table 1:** Chemicals associated with adverse MG effects, highlighting susceptible exposure windows

Effect	Model	Pre/perinatal (daughters/F_1_)	Peripubertal	Pregnancy/postpartum (maternal)	Adulthood
Lactation	Human			^b^PFAS (PFOA [7–9, 51], PFOS [7, 8, 51], PFNA [7, 8]), ^b^DDT/DDE [54–56], Smoking [46–50], ^b^PBB [57]	Pharmaceutical hormones [316, 317]
	Rodent	Atrazine [30, 60], BPA [62, 68], PFOA [58], TCDD [35], Ziracin [37]		Atrazine [59], BPA [61], BPS [39, 63], PFOA [33, 34, 58], TCDD [32, 64, 65]	
Accelerated puberty (thelarche)	Human	DES [86], BPA [91]	BPA [92], 2,5-dichlorophenol [93], triclosan [93, 94], 4-phenylphenol [94], methyl paraben [95], phthalates (MMP [108, 110], MEP [108], MBP [108, 318], DMP [111], DEP [111], DBP [111], DEHP [111] and its metabolites (MEHP [106, 108, 109], MEHHP [108, 109], MEOHP [108, 109]), DiNP [107], DiDP [107])		
	Rodent	Bisphenols (MG development: BPA [117–119], BPS [117], BPAF [117]; VO: BPA [113–116]), DES [121, 122], ethinyl estradiol [123], phthalates (MG development: BBzP [130], mix of DEP + DEHP + DBP + DiBP + DiNP + BBzP [129]; VO: DBP [133]), methyl paraben [66]	DES [120], genistein [120], *o,p'*-DDT [120], PFOA [126, 127], phthalates (VO: DEHP [134, 135], DBP [133]), methyl paraben [66], triclosan [66]		
Delayed puberty (thelarche)	Human	PCDD/F [96], PFOA [99, 102, 103], phthalates (MEHP [91], MBzP [105], DEHP [105])	^b^Dioxin-like compounds (unspecified) [97], ^b^DDE [98], hexachlorobenzene [98], benzophenone-3 [93], enterolactone [93], ^b^PCBs [98], ^b^PBDEs [98], phthalates (MBzP [112], DEHP [107])		
	Rodent	Atrazine [30, 60], TCDD [35], PFOA [33, 58, 124, 125], phthalates (MG development: DBP [132]; VO: DBP [131], DEHP [131], BBzP [130])	TCDD [120], PFOA [126], phthalates (MG development: BBzP [128]; VO: DEHP [135])		
Increased density	Human	DDT/DDE [186, 187]	BPA [184], MEP [184]		BPA [185], HRT [168–176], MEP [185], ^a^PAHs [188], ^a^PM_2.5_ [191], metals (^a^As [188], ^a^Co [188], ^a^Pb [188], ^a^Ni [188], ^a^Cr [188], ^a^Mn [188], ^a^Hg [188], ^a^Ni [188], Cd [189], Mg [190])
	Rodent	BPA [118, 193, 194, 198], DES [194], 2,4-dichlorophenol [196], PFOA [69],			2,4-dichlorophenol [196], o-nitrotoluene [197]
Cancer	Human	DDT/DDE [15, 213], DES [138]	^a^Air pollution/PAHs [219], ^b^DDT/DDE and other organochlorine pesticides [15]	^a^Air pollution/PAHs [219], ^b^DDT/DDE [15], ^b^PCBs [15, 17], ^b^PFOSA [15, 17, 216]	Pharmaceutical hormones [138, 213], ^b^TCDD [213], ^b^unspecified dioxin-like compounds [15, 17], ^b^PCBs [17, 213, 242], ^b^PBDEs [213], ^b^PFAS (PFOA [213] [216], PFOS [216], PFHxS [216], PFDA [216]), ^b^DDT/DDE [17], organophosphate pesticides [17], phthalates (MCPP [213], MCNP [213], MCOP [213], MEHP [213], DEHP [17, 213], DBP [17], DEP [17], MEP [17], MECPP [17]), ^a^Air pollution [15, 17, 215], PAHs [17], zearalenone [213], organic dyes [17, 226], metals (^a^Hg [17], As [17])
	Rodent	Bisphenols (BPA [139, 227, 232–234] (also sensitizes to DMBA [233, 237–239]), BPS [117, 229], BPAF [117]), PCBs [242] (also sensitize to DMBA and MNU [242]), TCDD sensitizes to DMBA [240], phthalate mixture sensitizes to MNU [129]	BPA sensitizes to DMBA [238]		Pharmaceutical hormones [138], PAHs [225], vinyl chloride [224], vinyl fluoride [224], organic dyes [226], PCBs (Aroclor 1254 and metabolite of PCB-61 induce tumors [242]; PCB-77 sensitizes to DMBA [241])

^a^Exposures were based on air pollution monitoring and individuals’ residences at the time of diagnosis. It was unclear how long participants had lived in the location, so the window and duration of exposure is unclear. ^b^Measurements of legacy chemicals (e.g., dioxins, PCBs, PBDEs, PFAS, DDT/DDE) may reflect early or continuous exposures and are not necessarily confined to the window indicated. Reviews of epidemiological studies [15, 17, 50, 138, 213, 215, 216, 224–226, 242, 317] also include articles showing no associations between exposures and effects. Notably, these often do not consider WoS

In the last decade, numerous epidemiological studies have reported significant relationships between higher maternal serum PFAS (including perfluorooctanoic acid (PFOA), perfluorooctane sulfonic acid (PFOS), and perfluorononanoic acid (PFNA)) during pregnancy and/or lactation and decreased breastfeeding duration or increased risk of terminating breastfeeding earlier than anticipated [7–9, 36, 51–53]. These well-powered studies were conducted in three countries and were consistent in women of multiple races and ethnicities, suggesting the effects are generalizable. In addition, data on the duration of breastfeeding were collected prospectively during lactation, decreasing the potential for exposure or effect misclassification that may occur in retrospective analyses.

A reduced duration of breastfeeding has also been associated with higher levels of certain chemicals in maternal serum, including dichlorodiphenyl dichloroethylene (DDE; the most stable metabolite of the pesticide dichlorodiphenyltrichloroethane (DDT)) [54–56] and polybrominated biphenyls [57]. Higher maternal DDE was also associated with a lower likelihood of initiating breastfeeding [56]. Notably, women with higher levels of DDE were likely to attribute early cessation of breastfeeding to insufficient milk supply, poor infant weight gain, or infant difficulty in breastfeeding [55].

### Experimental Studies

Because of inherent limits to epidemiological studies, experimental studies in animal models, typically rodents, provide a controlled way to investigate chemical effects on lactation in order to make informed decisions about whether widespread human exposure is a concern. In mice, developmental exposure to PFOA has been associated with dose-dependent postnatal mortality and morbidity, which the authors hypothesized to be a result of the mother’s lactational output [34]. In subsequent PFOA studies that collected and evaluated whole-mounted MGs and histopathological sections, the MGs of dams exposed in utero showed delayed morphogenesis compared to controls, resulting in stunted alveolar development and decreased milk protein gene expression when lactation should have been at its peak [33, 58]. In addition, when controls displayed the typical signs of involution at lactation day 20, PFOA-exposed mice had MGs that appeared similar to those of mice at peak lactation (typically lactation day 10), further suggesting a delay in MG differentiation due to PFOA exposure. This developmental delay in gestationally exposed dams corresponded with a decrease in pup birthweight and weight gain throughout lactation, with no other signs of maternal toxicity or altered litter size. Both chronic (gestational/lactational and continued through drinking water) and gestational/lactational exposure to PFOA impaired MG development in female offspring and delayed differentiation of the lactating gland during pregnancy across three generations of mice, raising concerns about multigenerational effects of PFOA exposure on lactation in humans.

Other chemicals with evidence of effects on lactation in rodents include atrazine [30, 59, 60], BPA [61, 62], bisphenol S (BPS) [39, 63], 2,3,7,8-tetrachlorodibenzo-p-dioxin (TCDD) [32, 35, 64, 65], triclosan [66, 67], and the pharmaceutical ziracin [37] (Table 1). These experimental studies have shown that exposures during pregnancy may lead to reduced pup weight gain, increased pup mortality and/or morbidity, reduced milk in stomach following a nursing event, increased amount of time nursing, impaired differentiation of the MG, or earlier MG involution compared to unexposed controls. For instance, gestational administration of atrazine significantly delayed MG epithelial growth in F_1_ offspring [30, 60]. This stunted MG development prior to pregnancy may have reduced milk production, resulting in reduced weight gain in the F_2_ generation during the nursing period [30]. Furthermore, exposing pregnant dams to atrazine, BPS, or TCDD altered prolactin levels and/or signaling [32, 39, 59]. BPS exposure to dams during pregnancy and lactation also reduced the fraction of milk-producing units in the MG and was associated with longer nursing events, decreased pup-initiated nursing, and stunted pup growth and development [39]. Both BPA and TCDD exposure in dams reduced white milk spots in the stomachs of pups and significantly reduced pup weight gain during lactation compared to controls, suggesting impaired milk production [32, 61], which was confirmed for TCDD by MG whole mount evaluation [32]. Finally, gestational and lactational exposure to BPA delayed MG differentiation in the F_1_ generation and altered milk composition and yield in F_1_ lactating dams [62, 68].

Lactational defects after prenatal chemical exposures may sometimes be referred to as pup toxicity (noted stunted growth, lethargy, small amount/lack of milk in stomach), but most study designs are not able to distinguish between effects on the mother (maternal toxicity) and indirect effects on the pups (likely exposed in utero and via maternal milk). Several approaches allow experimenters to determine if effects on pup growth are due to lactational deficits in the mother. One approach is to cross-foster pups, creating groups of gestationally exposed and unexposed offspring nursed by unexposed mothers, allowing weight gain to be compared across groups [32, 60, 69]. Unfortunately, cross-fostering experiments are labor-intensive and require more animals. Another approach is to assess milk production in a fraction of the lactating dams several days after birth, using controlled nursing periods as in Rayner et al. [30], but this approach is also labor-intensive and requires milk collection skills. A third way is to collect the MG from dams at weaning and compare whole-mounted MG morphology and histopathology to controls [64]. MG whole mounts enable comparison of lobuloalveolar/adipocyte fractions in the MG, and histopathological analyses can reveal effects on involution (apoptotic bodies and increased fat cells) and active milk production using traditional staining (milk lipid content) or immunohistochemistry (milk protein or apoptotic markers). Changes in milk proteins or apoptotic markers may also be quantified using collected tissues and qPCR or Western blot [32, 33]. Assessment of the dam MG morphology or biomarkers of lactational involution enables direct assessment of MG functional endpoints and involves the lowest time and cost investment compared to additional cross-fostering and controlled nursing experiments.

### Intersection of Epidemiological and Experimental Studies

To date, very few chemicals have been evaluated for lactation effects in both humans and laboratory animals. The similar effects of PFOA on lactation outcomes in rodents and humans are one important exception that increases confidence in the epidemiological findings. One historical criticism of lactational studies in humans, and possibly the reason that there are not more studies, has been the difficulty in separating physiologic reasons for early weaning (e.g., hormone inadequacy or undifferentiated breast tissue) from psychological or sociocultural factors, such as lack of familial support and maternal perceptions about low milk supply or quality [70]. Recent PFAS studies [7–9, 36, 51] have addressed those issues in cohorts of women committed to exclusive breastfeeding; the women donated serum during pregnancy (the time during which lobuloalveolar development begins) and had no knowledge of their environmental exposures before or during the study.

Experimental studies in rodents provide an alternative approach to studying chemicals’ effects on lactation, and they have the added benefit of flagging chemicals of concern before they can affect humans. Although there are some differences in the morphology and organization of the rodent MG compared to the human, rodents are sufficiently similar in the types of cells in mammary tissues and their sensitivity to numerous hormones [71, 72].

## Puberty

In recent decades, girls have been achieving pubertal milestones at increasingly earlier ages, with larger shifts in breast development (thelarche) compared to circulating sex hormone levels and age at menarche [4, 6, 73, 74]. Early thelarche has been associated with an increased risk of breast cancer [75, 76], heart disease [77, 78], obesity [79, 80], and psychological issues [81, 82]. A meta-analysis of 30 longitudinal or cross-sectional datasets from across the globe demonstrated a statistically significant decrease in age at onset of thelarche (Tanner stage B2) by 0.24 years per decade (*p* = 0.02) (Eckert-Lind et al. 2020). The decoupling of breast development from other aspects of puberty underscores that the two processes (thelarche and menarche) are regulated differently by hormonal signals [73, 83–85].

Although it is well established that in utero exposure to estrogens accelerates pubertal timing in both humans and experimental studies [16, 86], the intricate nature of hormonal influences on pubertal timing are still being discovered [83, 87, 88]. As one example, the authors of a prospective study of hormones and puberty observed that early breast development was more common in girls with higher adiposity and lower serum estradiol, and they hypothesized that early breast development was due to peripheral conversion of adrenal androgens to estrogens by aromatase in adipocytes [83]. A recent study that identifies 296 chemicals that increase estradiol or progesterone synthesis in vitro [14] suggests chemical exposures can affect pubertal development through aromatase metabolism, in addition to direct effects on estrogen and other hormone receptors [89, 90]. Since MG organogenesis begins during fetal development, EDC exposures during pregnancy have the potential to significantly affect both the mother and her offspring.

### Epidemiological Studies

Several epidemiological studies have linked exposure to EDCs with alterations in the timing of thelarche. For example, a large cohort study showed that maternal use of diethylstilbestrol (DES) and smoking during pregnancy were associated with early thelarche [86]. Higher levels of urinary BPA were also associated with early thelarche whether the exposure was measured in the mother during pregnancy [91] or in the peri-pubertal adolescent [92], as were the highest peri-pubertal levels of 2,5-dichlorophenol [93], triclosan [93, 94], 4-phenylphenol [94], and methyl paraben [95] (Table 1).

Other epidemiological studies have demonstrated associations between EDC exposures, as measured by blood or urinary levels, and delayed thelarche. Cohort studies have shown that perinatal exposure to polychlorinated dibenzo-p-dioxins and -furans (PCDD/F) [96] and peri-pubertal levels of dioxin-like and non-dioxin-like polychlorinated biphenyls (PCBs) and polybrominated diphenyl ethers (PBDEs) [97, 98], DDE [98], hexachlorobenzene [98], benzophenone-3 [93], and enterolactone [93] were associated with delayed thelarche (Table 1).

Additionally, prenatal [99] and peripubertal [100] exposure to PFAS chemicals have been associated with delays in menarche; while these studies did not monitor thelarche, the delay in pubertal timing may also have manifested in delayed breast development, since these two pubertal endpoints have been correlated in some populations [101]. One study suggesting that lactational exposure to PFAS alters timing of thelarche came from cohorts of ethnically diverse girls aged 6–8 from the New York City, Greater Cincinnati, and San Francisco Bay areas [102, 103]. In all three cities, breastfed or mixed-fed (breastmilk and formula) girls achieved Tanner stage B2 later than girls exclusively fed formula [103]. Since PFAS can be transmitted to infants through breastmilk, breastfed infants can be more highly exposed to PFAS than those fed formula [104], and indeed, in the Cincinnati and San Francisco cohorts, breastfeeding duration was positively associated with higher serum PFOA levels in the girls years later [102].

In some cases, the relationship between environmental chemicals and timing of thelarche is not straightforward. For example, the effect of phthalates on the timing of thelarche appears to depend on the window of exposure and the specific chemical (Table 1). Prenatal exposure to certain phthalates has been associated with delays in breast development [91, 105], whereas higher peripubertal levels of phthalates have usually been associated with earlier thelarche (with some exceptions) [106–111].

Most cohort and case–control studies show that peripubertal levels of high molecular weight phthalates (e.g., di(2-ethylhexyl) (DEHP), mono(2-ethylhexyl) (MEHP), diisononyl (DiNP), and diisodecyl (DiDP) phthalates) and their metabolites are associated with earlier thelarche [106–109, 111]. However, one large cohort study (*N* = 1170) showed a weak negative association between monobenzyl (MBzP) phthalate and breast development [112], and another cohort showed a negative correlation between DEHP and breast development [107]. These divergent effects highlight the importance of considering WoS and specific chemical substances in studies of environmental exposures and endpoints related to endocrine activity. On the other hand, the ostensibly conflicting evidence from studies of phthalates and thelarche timing may reflect (1) the challenge of obtaining a reliable long-term exposure estimate for rapidly-metabolized phthalates, or (2) the mixture of EDCs present in many phthalate exposure sources, particularly since behavior changes associated with puberty (e.g., cosmetics, perfume use) may increase certain phthalate exposure biomarkers as well as other EDC exposures.

### Experimental Studies

Numerous studies have documented the effects of in utero and early life exposure to environmental chemicals on the timing of puberty in rodents. For example, either prenatal or postnatal exposure to BPA accelerated puberty (as indicated by vaginal opening) in rodents [113–116]. Prenatal exposure to BPA, BPAF, or BPS also caused precocious MG epithelial growth and branching [117–119], with two of these studies showing a non-monotonic dose–response [117, 118]. In other examples, exposure to diethylstilbestrol (DES; perinatal or peripubertal), ethinyl estradiol (perinatal), genistein (peripubertal), *o,p*’-DDT (peripubertal), methyl paraben (perinatal or peripubertal), or triclosan (peripubertal) accelerated MG proliferation and differentiation [66, 120–123]. On the other hand, prenatal or peripubertal exposure to TCDD in rats delayed the development of ductal epithelial branching and budding in the MG [35, 120] as did prenatal exposure to the high-use pesticide atrazine [30, 60].

Experimental studies have explicitly demonstrated the importance of WoS and dose levels for effects on pubertal timing. For example, prenatal PFOA exposure delayed MG development in mice [33, 58, 69, 124, 125], whereas peripubertal exposure could accelerate or delay MG development with strain- and dose-dependent differences [126–128].

The effects of phthalates on pubertal indices in rats also vary with WoS, dose, and chemical species. Perinatal exposure to higher molecular weight phthalates tended to increase MG proliferation and differentiation [129, 130] but interestingly, such exposures tended to delay vaginal opening [130, 131]. On the other hand, perinatal exposure to dibutyl phthalate (DBP; low molecular weight) in rats led to hypoplasia and poor ductal branching in the pubertal female MG, as well as nipple retention and MG degeneration in young and adult males, respectively [132]. Postnatal exposure to lower doses (50 mg/kg/d or less) of phthalates reduced terminal end buds and proliferation in the MG by the end of puberty [128] but accelerated vaginal opening [133–135], whereas exposure to a very high dose of DEHP delayed vaginal opening [135].

The divergent effects of phthalates on vaginal opening and MG development in rodents may be due to differences in endocrine pathways involved in MG development vs. other aspects of puberty, in addition to the specific windows of exposure and chemical characteristics. For example, phthalates’ effects on male reproductive development have been attributed to altered steroidogenesis (reduced fetal testosterone) through modes of action that may also be important for MG development, such as steroidogenic factor-1 [136, 137]. A better understanding of mechanisms for endocrine disruption of MG development and function would allow for development of more comprehensive chemical screening programs that interrogate these mechanisms, facilitating prioritization of chemicals for further study.

### Intersection of Epidemiological and Experimental Studies

Many of the chemicals associated with alterations in thelarche timing in humans have also been shown to alter the timing of puberty in the same direction, and with similar WoS, in animal studies. Species concordance is evident for accelerated breast development by BPA (perinatal and peripubertal in humans [91, 92], perinatal in rodents [117–119]); methyl paraben (peripubertal in humans [95]; perinatal and peripubertal in rodents [66]); and triclosan (peripubertal in humans [93] and rodents [66]); and for delayed development by dioxin-related compounds (prenatal and peripubertal in both humans [96–98] and rodents [35, 120]) (Table 1).

Remarkably, prenatal exposure to higher molecular weight phthalates is associated with delayed thelarche in humans [91, 105] and delayed vaginal opening in rodents [130, 131], but pubertal development of the rodent MG appears to be accelerated by prenatal exposure to high molecular weight phthalates [129, 130] and delayed with low molecular weight phthalates [132]. Conversely, peripubertal levels of phthalates in humans are usually associated with earlier thelarche [106–111], and postnatal exposure in rodents accelerated vaginal opening [133–135]. While few studies have evaluated the effects of postnatal exposure to phthalates on MG development in rodents, one study in rats showed that peripubertal exposure to low doses of a high molecular weight phthalate reduced terminal end bud formation and epithelial proliferation in adults [128].

Overall, given that peripubertal levels of different phthalates can be associated with earlier or later thelarche in humans [106–112], and that exposing rodents to different phthalates during different WoS yields divergent effects on MG development, additional studies of phthalates’ effects on MG development and related signaling mechanisms in rodents could help clarify these seemingly contradictory results or reveal non-monotonic dose responses.

These observations that chemical exposures affect MG development underscore the importance of incorporating MG assessments into toxicological evaluations of development. In humans, early thelarche is more strongly correlated with breast cancer risk compared with other indicators of puberty (e.g., menarche or sex hormone production) [4, 75]. Chemicals that accelerate MG development in rodents also increase mammary tumors (e.g., DES [120, 138], bisphenols [117, 118, 139, 140]) so measuring this endpoint is critical in order to detect those risks. Additionally, evidence indicates that prenatal exposures to certain environmental chemicals can alter MG development and subsequently cause deleterious effects on lactation, as suggested by rodent studies with BPA [62, 68], PFOA [58], TCDD [35], and atrazine [30, 60]. Finally, regardless of subsequent effects on neoplasms or lactation, altered MG growth and development is an endpoint of concern for chemical risk assessment, and so chemicals that induce these changes should be identified (discussed further below).

## Mammary Density

Epidemiological studies also suggest that environmental exposures are associated with increased mammographic density (MD; the ratio of fibrous and glandular breast tissue to the total amount of breast tissue) (Table 1). MD has important implications for breast cancer risk, as it is a well-established risk factor for breast cancer [141–143] and it can mask tumors during mammogram screenings [144, 145]. Studies suggest that women with high MD can have a greater than four-fold higher risk of breast cancer than those with low MD [142, 146, 147]. Women with higher MD have also been reported to experience more aggressive breast tumors, potentially due to later tumor detection [148] or a more permissive environment for tumor growth [149–152].

Markers of breast density, including increased collagen density [153–155], tissue stiffness [156], and increased epithelia and corresponding periductal stroma [157], may be causally linked to tumor initiation and progression, as evidenced by both experimental [158–160] and epidemiological studies [161–163]. For instance, experimental studies using mice suggest that tumorigenesis is promoted in the presence of increased collagen [158] and extracellular matrix stiffness [160, 164–166], which collagen may also promote. Other studies have hypothesized that breast density reflects increased epithelial and stromal cell quantity and/or proliferation [153, 154, 157, 167], and the interaction of epithelia with stroma may promote epithelial-mesenchymal transitions [149–151].

### Epidemiological Studies

Perhaps the best-documented examples of changes in breast density by external agents in humans is from the use of hormone replacement therapy (HRT) and the drug tamoxifen in relation to breast cancer treatment. HRT has been shown to increase breast density [168–170] and breast cancer risk [171–176]. In fact, even a single year of treatment with estrogen plus progestin HRT was sufficient to increase MD as well as breast cancer risk [176]. In contrast, the selective estrogen receptor (ER) modulator tamoxifen [177–180] and stopping the use of HRT [169, 171, 181] decreased breast density and/or the risk of breast cancer. Tamoxifen is an ER antagonist in the breast, and it is sometimes prescribed to reduce risk among women with higher risk of breast cancer, including in women with dense breasts, and it is a first-line treatment to prevent breast cancer recurrence [180, 182]. These associations provide compelling evidence for the importance of estrogen and progesterone in breast density and cancer [183]. As such, chemicals that increase estrogen and progesterone biosynthesis may also increase breast density and, therefore, cancer risk [14].

Using breast imaging techniques such as mammograms, epidemiological studies have associated higher serum or urinary concentrations of several environmental chemicals with higher breast density, including BPA [184, 185], DDT [186, 187], polycyclic aromatic hydrocarbons (PAHs) [188], metals (e.g., cadmium, magnesium, nickel) [188–190], mono-ethyl phthalate (MEP) [184, 185], and air pollution [191] (Table 1).

### Experimental Studies

There are no standardized methods or tests to measure chemical effects on MG density in rodents, so guideline toxicology studies unfortunately do not assess MG density. However, there are related MG endpoints that have been assessed in hypothesis-driven experimental toxicology studies that include alterations in mammary fibrosity or opacity, gland “whitening” or “stiffening,” stromal collagen density, collagen matrix permeability, stromal or epithelial hyperplasia, and alterations in the size of the epithelial or stromal compartments (e.g., an increased number of mammary ducts, stromal hyperplasia, and density of Masson’s trichrome staining) [118, 192–195]. Some of these endpoints may also reflect changes in aspects of mammary development, and additional research is needed to better understand those relationships.

Experimental studies in rodents have reported increased stromal markers of MG density, described above, following prenatal administration of BPA [118, 194], DES [194], 2,4-dichlorophenol [196], and PFOA [69] (Table 1). Markers of MG density were also increased in adult rodents treated with 2,4-dichlorophenol [196] and o-nitrotoluene [197]. Interestingly, prenatal administration of BPA increased collagen deposition, number and diameter of type I collagen fibers, and MG stiffness, and decreased collagen matrix hydraulic permeability in adult 12-week old mice but not in 4-week old mice [194]. Changes in the periductal stromal composition have been observed during fetal development after BPA exposure, suggesting that these outcomes have an early developmental origin [193, 198]. Similar to BPA, a relatively long latency in mammary density changes was noted in mice developmentally exposed to PFOA [69]. In this study, mice exposed to PFOA in utero or in utero and via lactation had a less developed ductal epithelium in young adulthood, but they exhibited significant epithelial and stromal hyperplasia compared to controls at 18 months of age.

### Intersection of Epidemiological and Experimental Studies

More robust methods for measuring and reporting breast density in women could aid in identifying environmental agents that alter MD and the associated features that contribute to breast cancer. For example, research linking clinically reported MD scores (now mandated in at least 24 states [199]) with monitoring of environmental chemicals through the Environmental Protection Agency’s (EPA) National Air Toxics Assessment [200] has permitted measurement of epidemiological associations between environmental exposures and MD in large populations [188, 201]. Similar use of environmental chemical monitoring data, such as those gathered through the Unregulated Contaminant Monitoring Rule for drinking water and the Agency for Toxic Substances and Disease Registry’s PFAS Multi-Site Study, could enable epidemiological associations between chemical exposures in water and MD, as well as lactation, puberty, and breast cancer. In addition, imaging techniques that can be applied on a broader population (e.g., by using less radiation than mammography [202, 203]) or that provide more sensitive measures of density can facilitate investigation of relationships between MD and exposures.

Alternative breast imaging techniques—such as magnetic resonance imaging, ultrasound tomography, or digital tomosynthesis (3D mammograms)—may offer more accurate breast density measurements than standard mammography [202, 204]. Unlike standard mammograms, which compress features of the breast to create 2D images, other imaging techniques can estimate volumetric breast density, potentially providing a more accurate estimate of the fibroglandular tissue in the breast [202]. Importantly, these methods may shed light on the specific features of MD associated with increased risk of breast cancer. For example, tissue stiffness is a feature of breast density associated with increased breast cancer risk, but a standard mammogram alone may not be sufficiently sensitive to detect it [163]. Indeed, DES (a mammary carcinogen in humans and rodents [138]) is not associated with increased MD [205], but it increases mammary tissue stiffness in prenatally exposed mice [194], highlighting the need for additional studies to assess whether mammary tissue stiffness is a mechanism by which DES increases breast cancer risk in women.

Methods for measuring MG density and morphology in rodents and application of those methods in regulatory toxicology studies are also crucial for measuring impacts of environmental exposures. In order to collect robust information on chemicals that alter mammary histology and breast density, longitudinal sections of mammary tissue representing a large fraction of the gland are needed (Fig. 1a). These provide superior information to assess stromal changes and epithelial hyperplasia compared to the more typical transverse cross-section of tissue on skin [206]. Additionally, recent work [207] has demonstrated the utility of sectioned rat MG whole mounts for use in RNAScope evaluation; stromal density and stromal-specific genes could be quantified using this approach to better understand chemical effects.

## Breast Cancer

While cancer diagnoses have generally decreased over the last few decades, breast cancer stands out as one of the few cancers that is increasing in prevalence. The breast is now the most common site of new cancer diagnoses worldwide [12] and in the USA [208]. Moreover, among both women and men under age 50 in the USA, female breast cancer is the leading type of cancer diagnosis (2.5-fold higher than the next most common cancer in women, thyroid, and 5.5-fold higher than the most common in men, colon) as well as the leading cause of cancer death (over twofold higher than the next most common, male colon) [209]. Importantly, between 2000 and 2018, US breast cancer incidence in women under 40 years of age rose at a significant rate of 0.6% per year [11]. Abundant evidence indicates that genotoxic and hormonal exposures increase risk of breast cancer [138, 173, 210], and that such exposures during development can be more carcinogenic than during adulthood [15, 211, 212].

### Epidemiological Studies

The long latency of disease and challenges in assessing lifetime chemical exposures have limited the ability for epidemiological studies to assess relationships between exposures and breast cancer in humans. However, chemicals that alter normal MG development and function in rodent studies have also been detected in blood and urine of women, and those biomarkers of exposure have been associated with breast cancer risk. Recent systematic reviews highlight strong epidemiological evidence showing that DDT and its metabolites, TCDD, air pollution (e.g., PAHs, NO_2_, PM_2.5_, and heavy metals), organic solvents, and pharmaceutical estrogens (e.g., DES) are associated with increased risk of breast cancer, with some increasing the risk of premenopausal breast cancer, which is less likely to be confounded by age-related risk or demographic factors [15, 17, 213–215]. In addition, estrogen + progestin HRT after menopause has also been shown to increase breast cancer risk [138]. More limited evidence suggests associations between breast cancer and exposure to certain PFAS, PCBs, PBDEs, oral contraceptives, phthalates, hair dyes, and organophosphate pesticides [15, 17, 213, 216]. Increasing breast cancer rates in women who are too young to be recommended for yearly mammograms (< 40 years old) lend further support to the hypothesis that early life exposures can be carcinogenic [11, 17, 217–222]. Many of these chemicals associated with increased breast cancer risk have also been reported to have effects on lactation, MG development, and MD, having EDC properties that likely contribute to these effects [14, 15, 17].

### Experimental Studies

More than 200 chemicals cause mammary tumors in rodent cancer studies [23], suggesting they may also increase human breast cancer risk. These include carcinogens that act through genotoxicity, endocrine disruption, and a combination of these and other modalities associated with carcinogens [223]. A few well-established chemical classes of rodent mammary carcinogens include pharmaceutical estrogens, PAHs and nitro-PAHs, halogenated solvents, vinyl halides, and organic dyes [23, 138, 224–226].

Rodent studies also provide evidence that chemicals that alter MG development can also induce mammary tumors and/or sensitize the MG to other chemical carcinogens. One example is BPA, which, as noted above, alters MG development in rodents with exposure during WoS [118, 119, 227–231], and exposures during multiple WoS induce mammary hyperplasia and spontaneous mammary adenocarcinomas in rodents [139, 227, 232–235]. BPA analogs such as BPS and BPAF have been reported to have similar developmental and tumorigenic outcomes [117, 229]. Based on evidence that developmental exposure to BPA induces intraductal hyperplasia and tumors that manifest during adulthood, it meets the US Environmental Protection Agency’s (EPA) definition of a carcinogen [236] (i.e., BPA is a complete carcinogen). Prenatal BPA exposure also sensitizes the rodent MG to other mammary carcinogens, such as 7,12-dimethylbenz(a)anthracene (DMBA) [237–239].

Developmental exposure to TCDD and related chemicals (which affect lactation [32, 64, 65] and MG development [35, 120]) also sensitize the rodent MG to other carcinogenic exposures [240, 241], and some PCB mixtures are capable of inducing mammary tumors as complete carcinogens [242]. Other rodent mammary carcinogens that affect MG development and function include atrazine and pharmaceutical estrogens [22, 23].

### Intersection of Epidemiological and Experimental Studies

Chemicals that cause mammary tumors in animal studies may be considered likely human breast carcinogens based on mechanistic and/or tumor site concordance between humans and rodents [22, 23, 243, 244]. For example, both laboratory and human evidence support a role for chemicals that (1) alter MG development or hormone responsiveness, (2) increase hormonal activity, and/or (3) are genotoxic to promote mammary tumors [15, 210, 245]. These simplified pathways provide a helpful framework for considering exposure-effect relationships in both experimental and epidemiological studies of chemically induced breast cancer because they highlight important WoS and encourage consideration of whether any particular epidemiologic or experimental study design is matched to underlying biological processes. The lengthy and hormone-dependent developmental trajectory of the breast—which does not fully differentiate until lactation and undergoes many cycles of morphogenesis and regression—is likely to be a major factor in its susceptibility to cancer.

Of the more than 200 chemicals shown to induce mammary tumors in rodents [23], few have been studied adequately in humans [15, 22], highlighting the need for additional research into chemicals that can increase breast cancer risk. Note that since most cancer bioassays do not include early life exposure, tests may have missed potential breast carcinogens that act by altering early MG development, especially via endocrine pathways.

Conversely, studies of EDCs—which often include developmental exposures—are rarely carried out with the lengthy follow up and large numbers of animals needed to detect an increase in mammary tumors. Similarly, the long latency for breast cancer in humans following developmental exposure has been a barrier to studying associations [212, 218, 219, 221, 246]. As an example, epidemiological studies have not linked BPA exposure with breast cancer, but the case–control studies that have been conducted have not been able to reliably estimate early life exposures [247–250]. However, as discussed above, chemicals such as BPA and its analogs both alter MG development and induce hyperplasia and tumors in rodents that manifest during adulthood [117–119, 139, 227–234], demonstrating a plausible and direct connection between altered MG development and cancer.

## Cross Cutting Issues

Normal development and function of the breast is key to successful reproduction and offspring survival in mammals, and epidemiological studies have limited ability to pinpoint adverse effects from ubiquitous EDCs and their mixtures. Thus, it is essential that toxicological studies collect and evaluate the MG consistently and effectively to identify chemicals that affect breast outcomes identified here. Many critical gaps in current practices for chemical toxicity testing and risk assessment limit our ability to identify and prevent exposures that may adversely modify breast development and function.

Increased understanding of EDCs in recent decades has led to modernization of toxicological test methods to include exposures during WoS and new hormone-sensitive endpoints, but unfortunately, testing of the MG remains inadequate. For example, several testing approaches detect chemicals that affect androgen, estrogen, thyroid, and steroidogenic (EATS) mechanisms of action [251], and some have argued that these modes of endocrine disruption are sufficient to detect chemicals that will harm the MG. However, multiple additional pathways influence MG development and function that are not necessarily captured by standard endocrine-sensitive endpoints (e.g., vaginal opening, estrous cyclicity, serum hormones, reproductive organ weights). For example, multiple stages of MG development are influenced by the aryl hydrocarbon receptor [252], progesterone [2, 253], prolactin [254–256], insulin-like growth factors [253, 255, 257, 258], transforming growth factor-α [259], and placental lactogens [260]. While some of these factors are also involved in other aspects of reproductive development [261–265] and may be detected in other typically measured endpoints, the interplay of these mechanisms and magnitude of effects likely differ between tissues. As one example, hormone levels are typically measured in serum, which does not account for peripheral steroidogenesis in mammary tissue, so chemicals that alter local steroidogenesis are not detected with serum hormone levels. Alterations to functions that are specific to the MG cannot be detected unless the MG itself is assessed.

Many chemicals that affect MG development act via these (and perhaps other) diverse pathways, including BPA [119, 266], atrazine [14, 267], TCDD [268], phthalates [137], PFOA [269, 270], and PBDEs [271]. However, regulatory risk assessments often fail to consider these effects because the MG is often not examined sufficiently to detect them. This is particularly important because effects on the rodent MG can be detected at doses well below those affecting more traditional endpoints for evaluating endocrine disruption, such as age at vaginal opening, estrous cyclicity, circulating sex hormone levels, and uterine and ovarian weights [16, 117, 125, 128, 132, 272]. Since these traditional endocrine endpoints do not reflect the sensitivity of the mammary tissue, results can be misleading for risk assessment, and can lead regulatory agencies to inaccurately conclude that evaluation of MG effects is not needed or that a chemical has no effect on the MG. Considering the totality of the evidence, it is urgent to strengthen the toxicological evaluation of mammary biology spanning in utero development through early tumorigenesis. Below, we highlight several of these issues and provide recommendations.

### Tissue Evaluation in Rodent Toxicology Studies

Despite the growing body of epidemiologic evidence demonstrating effects of environmental exposures on the breast, as summarized above, current guidelines (i.e., requirements) for toxicity testing required by regulatory agencies or conducted by governmental research centers are vague and/or incomplete regarding MG collection and evaluation (Table 2). In this section, we identify reproductive/developmental and carcinogenicity testing requirements from the Organisation for Economic Cooperation and Development (OECD) (followed by the European Chemicals Agency, US National Toxicology Program (NTP), and US EPA Office of Chemical Safety and Pollution Prevention (OCSPP) that may be best able to capture effects of exposures during development or lactation and identify later life effects from breast toxicants. We provide a brief overview of guidance documents for pharmaceutical testing developed by the International Council for Harmonization of Technical Requirements for Pharmaceuticals for Human Use (ICH) that are used by the US Food and Drug Administration (FDA) and European Medicines Agency. We also describe additional pathology guidance for assessment of the MG as suggestions for improvement.

**Table 2:** Regulatory toxicology test guideline elements for detecting adverse effects on the mammary gland

Study design element	Key purpose/importance	Recommendation	Reproductive/developmental guidelines			Carcinogenicity guidelines		
			OECD [273, 280]	US NTP [274]	US EPA [275–279]	OECD [284]	US NTP [283]	US EPA [285]
Pre/perinatal exposure	Most sensitive windows for EDC effects	Include for all guideline studies	Yes	Yes	Yes	No	Sometimes	No
Developing MG assessment	Altered development reflects teratogenicity	Include for all developmental exposures	No	Yes	Not mentioned	*NA: no developmental exposure*	No	*NA: no developmental exposure*
Adult MG assessment	Detect persistent/long latency effects	Always	Yes	Yes	Not mentioned	Yes	Yes	Yes
Assessment of all dose groups	Detect non-monotonic dose responses (common for EDCs)	Always	All F_1_ adults; P_0_ if effects observed at high dose	Control and high dose, others as needed to establish no-effect level, animals dying before study end	*NA: MG not mentioned*	Macroscopic lesions, if effects observed at high dose, animals dying before study end	Yes	Macroscopic lesions, if effects observed at high dose, animals dying before study end
Male MG assessment	Sensitive to EDCs	Always	All F_1_ adults; P_0_ control and high dose, and if effects observed at high dose	Language is included for histology from both sexes	*NA: MG not mentioned*	If “visibly dissectible”	Yes	No
Histopathology	Detect microscopic lesions	Longitudinal cross-sections for all animals	Transverse cross-section (control and high dose, other doses if triggered)	Longitudinal cross-section (control and high dose, other doses if triggered)	*NA: MG not mentioned*	Transverse cross-section (control and high dose, other doses if triggered)	Transverse cross-section	Section type not indicated (control and high dose, other doses if triggered)
Whole mount analysis	Observe ductal branching and detect early lesions	Always	No	Optional	*NA: MG not mentioned*	No	Optional	No

#### Reproduction and Developmental Studies

To identify early life developmental and functional MG deficits, the tissue should be evaluated in guideline or fit-for-purpose studies with gestational/lactational test article exposures. There are only a handful of guideline studies that can address these effects, and they have limitations in their ability to detect effects on the MG (Table 2).

A limitation in the OECD extended one-generation reproductive toxicity study (EOGRTS; TG 443) is that not all MGs from all groups are assessed. MG histopathology is required for all F_1_ adults (male and female), but in parental animals, full histopathology of the MG is initially performed only for high-dose and control [273]. MGs from low- and mid-dose parental animals are only assessed if changes are observed at the high dose, as OECD TG 443 states that, “organs demonstrating treatment-related changes should also be examined in all animals at the lower dose groups to aid in determining a NOAEL” [273]. Since EDCs can induce significant changes at lower doses [58, 117, 118, 140], omission of lower dose groups can miss these effects.

US agencies also have limitations for MG assessments in their reproductive and developmental toxicity testing. The NTP Reproductive Assessment by Continuous Breeding (RACB) and Modified One-Generation (MOG) specifications [274] require complete histopathologic evaluation on all control and high dose groups, lower dose groups as needed to establish a no-effect level, and animals that die or are sacrificed before the study end. Currently, these specifications also contain a description of MG whole mount collection and evaluation (as of June 2022) and state that they should be assessed “if required” in addition to histopathology, but without further elaboration on conditions for requirement. However, these specifications are being replaced with fit-for-purpose approaches and it is unclear if the MG will be evaluated at all. Additionally, the word “mammary” does not appear in EPA’s Test Guidelines for Pesticides and Toxic Substances for reproduction and developmental toxicity [275–279], suggesting that they are likely not being collected or evaluated.

Finally, while NTP and OECD reproductive and developmental toxicity test guidelines include endpoints that may help identify lactational deficits (e.g., pup growth and survival) if they exist [273, 274, 280], the study designs cannot distinguish between maternal lactation insufficiency and pup toxicity, as the maternal MG is not assessed until after weaning (if at all). Assessment of the maternal MG during lactation, using a fit-for-purpose approach, would be needed to provide important information about lactational deficits as well as context for any observed toxicity in pups. We recommend greater attention to pup weight differences between birth and postnatal day (PND) 4 (culling) as a potential indicator of a lactation effect.

ICH S5 guidelines for developmental and reproductive toxicity testing of pharmaceuticals do not address possible effects on the MG [281]. This guideline describes three study designs to address fertility and embryonic, fetal, and postnatal development, and none requires collection of the MG from dams or pups unless macroscopic lesions are visible, thereby precluding the possibility for detecting effects on MG morphogenesis or lactation. The ICH and FDA indicate that these guidelines are flexible and can be adapted at the discretion of the sponsor [281, 282], so it is unclear which endpoints are consistently required for assessing maternal and pup toxicity from pharmaceuticals.

#### Carcinogenicity Studies

The major limitations of carcinogenicity test guidelines are the lack of exposure during relevant WoS, the reliance on gross observations for MG collection or assessment, and the method by which MG tissue is collected and analyzed (Table 2).

Historically, OECD, EPA, and NTP carcinogenicity guideline studies initiated dosing in adult animals and not during development [283–285]. Recently, NTP has begun to expose animals (rats only) from gestation through lactation and post-weaning in cancer bioassays [286–289]. As a result, older cancer bioassays may have missed carcinogenic effects of EDCs that alter MG development. For risk assessments using older data, uncertainty factors could be applied to address lack of developmental exposures. In the future developmental exposures should be included in carcinogenesis studies for all potential EDCs.

In terms of triggers for assessing the MG, OECD and EPA OCSPP carcinogenicity test guidelines require histopathology of the female MG for all animals that died or were euthanized early during the study; when macroscopic (gross) lesions are observed; in control and high-dose animals; and other dose groups if treatment-related lesions are observed in the high-dose group [284, 285]. Thus, in addition to typically lacking developmental exposures, these carcinogenicity studies frequently do not perform histopathology on low- and mid-dose groups, and may have reduced numbers of animals in some groups for terminal comparison purposes if early terminations exist in some groups and not all.

The ICH S1B guidelines for testing pharmaceuticals for carcinogenicity do not list any tissues that are required for assessment, and these guidelines are also fit-for-purpose depending on the type of drug [290, 291]. Presumably when carcinogenicity studies are conducted on pharmaceuticals, the MG would be assessed at least if macroscopic lesions are present, but no language in the guideline document indicates such a requirement.

#### Non-Monotonic Dose-Responses

A limitation of some of the aforementioned study designs is that tissues from low- and mid-dose groups may only be required when treatment-related lesions are observed at the high dose (Table 2). However, some chemicals produce different effects on the MG at low compared to high doses, as has been illustrated following developmental exposures to BPA and PFOA [58, 117, 118, 140, 235]. The non-monotonic response of the MG to estrogenic chemicals is well demonstrated [230, 231, 292–294], and thus could be assumed when interpreting data from test guideline studies. Furthermore, statistically significant induction of human-relevant mammary tumors at lower doses has sometimes been dismissed because high dose groups with reduced body weight developed equivalent numbers of tumors as controls [235, 295], despite evidence that reductions in body weight decreases the propensity for mammary tumor induction [296, 297]. As these examples demonstrate, the requirement for a monotonic dose–response is not indicated in the case of EDCs. Instead, mammary tissues from all dose groups should be collected and analyzed, and significant differences from control should be determined at each dose.

#### Male Mammary Gland Assessment

Although much attention has been devoted to the female MG, incidence of breast cancer and gynecomastia has also increased in males [298], and studies in rodents indicate that the male MG is highly sensitive to EDCs [231, 299–305]. To understand the environmental contribution to these population trends, toxicity test guidelines should require collection and histopathology of the male MG. Currently, histopathology of the adult male MG (F_1_ and parental) is part of the OECD EOGRTS guideline [273] and required for OECD carcinogenicity testing if “visibly dissectible, from males” [284]. Notably, the OECD carcinogenicity test guideline does not specify whether the male MG should be collected if a *lesion* is visibly dissectible, or if the gland itself is dissectible; the MG itself is always dissectible, but the vague language implies that collection of the male MG is optional. While the NTP chronic exposure carcinogenicity test specifications require histopathology of the male and female MG [283], NTP developmental toxicity specifications are less descriptive but include language for histopathology of the MG from both sexes [274]. It is thus unclear if or when developmental data would be generated on the male MG. As noted above, EPA OCSPP reproductive and developmental guidelines do not mention MG assessment [275–279], and the carcinogenicity guideline only includes assessment of the female MG [285].

#### Tissue Collection and Assessment Methods

Historically, MG analyses in toxicological studies have been performed using transverse cross-sections including skin. However, these histologic sections capture only a small amount of MG ductal tissue (Fig. 1a). A suggested improved approach is to collect the entire 4th gland in rats, with the 4th and 5th collected in mice, and evaluate longitudinal sections that provide greater coverage of the MG (Fig. 1a), as advised for toxicologic pathologists [206].

A more comprehensive approach to identify chemical effects on MG morphology and pathology is to use MG whole mounts complemented with histology sectioning, as is required by NTP RACB and MOG specifications when the MG is included [274] and suggested (but not required) in the OECD EOGRTS guideline [280]. EPA OCSPP carcinogenicity guidelines [285] indicate that histopathology of the female MG should be analyzed (see “Carcinogenicity Studies” section for conditions for assessment), but there is no guidance on how the section should be cut, and whole mount is not mentioned.

Whole mount analysis in reproductive- and younger-aged rodents is an inexpensive and straightforward method that provides a complete view of the 3D MG epithelial structure, allowing for detection of lactational morphology deficiencies (Fig. 1b), early and persistent developmental abnormalities (Fig. 1c), microscopic lesions such as ductal beading (intraductal hyperplasia or carcinoma in situ) (Fig. 1d), inflammatory infiltrates, and early tumor development (Fig. 1e) [21, 30, 32, 33, 35, 39, 58, 60, 69, 117–119, 123–127, 139, 193, 227, 229, 232, 305–308]. Whole mounts improve the quality of MG morphological analysis, adding information such as extent of ductal branching and differentiation. The tissue can subsequently be sectioned to evaluate microscopic lesions, decreasing the possibility for false-negatives that result from missing microscopic lesions when selecting histological sections to inspect [309]. Moreover, adding evaluation of MG whole mounts to test guidelines can provide information on developmental abnormalities or adverse outcomes not detected in other tissues without using additional animals in separate studies. Despite over 20 years of studies demonstrating that the whole mount approach is effective for detecting microscopic alterations in the MG, guideline specifications have yet to routinely adopt this technique.

In addition to the way the MG is collected and sectioned, it is important to have standardized approaches for histopathological analyses. The International Harmonization of Nomenclature and Diagnostic Criteria for lesions in rats and mice (INHAND; [310]) was developed by international toxicologic pathologist societies to diagnose lesions using harmonized diagnostic criteria and terminology. However, neither the INHAND criteria nor the OECD guidance on MG histopathology [311] address non-neoplastic and developmental changes that can be observed with whole mounts and MG sections.

In sum, we recommend that toxicological guideline studies be updated to specify that the MG be collected and analyzed (1) from all dose-groups, (2) at all time points, (3) from both females and males, and (4) with longitudinal histological sections complemented with whole mounted tissue. We also emphasize the importance of considering key WoS in study designs to capture effects on the MG that do not occur with adult exposures. These recommendations would enhance not only reproductive/developmental and carcinogenicity studies but also any study design for which the MG could be a target.

### Exposure Timing and Windows of Susceptibility

An important approach for identifying effects of EDCs on the developing MG is to query key WoS. The MG undergoes dynamic developmental processes for longer than any other tissue, with distinct biological alterations during gestational, perinatal, pubertal, pregnancy/lactation, and menopausal periods [3, 18, 19, 312]. Chemical exposures, particularly hormonally active ones, can exert adverse effects during any of these WoS, and early developmental alterations can predispose the tissue to adverse outcomes later in life (e.g., reduced ability to breastfeed and cancer, as described above) [3, 18, 19, 234, 313]. The chemical effects on the MG during WoS discussed in the previous sections are summarized in Table 1. While targeting particular WoS has become more common in certain laboratories, public health would benefit from standardized regulatory test guidelines that incorporate relevant WoS.

Crucially, the OECD, NTP (with recent exceptions for sodium tungstate, PFOA, DBP, and DEHP [286–289] and upcoming reports on black cohosh and tris(chloropropyl) phosphate), and EPA 2-year cancer bioassays do not capture some of the most relevant sensitive exposure periods for breast cancer (i.e., in utero, perinatal, and pubertal) because they start dosing after these WoS [283–285]. In contrast, these agencies’ developmental and reproductive toxicity guidelines capture in utero exposures and could thus provide key information about adverse impacts on MG development in early life that may predict later life effects if the MG were assessed [273–279]. Furthermore, prenatal exposures may have multigenerational effects on the MG [30, 58], but there are few test guidelines in which this type of assessment might be made.

A high-priority gap in testing guidelines is that the most common guideline used to detect effects of EDCs—the OECD EOGRTS—does not require assessment of MG effects in weanlings or other young adult endpoints. Specifically, the OECD EOGRTS test guideline recommends that pups not selected for cohorts are terminated after weaning on PND22, unless results indicate the need for further in-life investigations. The guideline suggests that, from up to 10 pups per sex per group, “mammary tissues for these male and female pups *may be preserved* for further microscopic analysis^1^ (see GD 151 (40)). Gross abnormalities and target tissues should be saved for possible histological examination,” with the footnote “Research has shown the mammary gland, especially in early life mammary gland development, to be a sensitive endpoint for estrogen action. It is recommended that endpoints involving pup mammary glands of both sexes be included in this Test Guideline, when validated” [273]. As of yet, MGs from weanlings have not been assessed in EOGRTS testing, as examination methods have yet to be validated. Validation of whole mount and other methods, and updating the requirements for the OECD EOGRTS, is critically needed in order to identify EDCs that affect MG development at key WoS.

### Altered Mammary Gland Structure and Developmental Timing Represents a Teratogenic Effect

Beyond gaps in MG assessments in toxicology studies, it is essential to consider how MG effects are considered in risk assessment, when they are reported. Specifically, there has not been a consensus about whether altered morphology and timing of MG development should be considered an adverse developmental effect [16], a.k.a. a teratogenic effect. Teratogenic is defined in the US EPA Integrated Risk Information System (IRIS) Glossary as “structural developmental defects due to exposure to a chemical agent during formation of individual organs” [236]. Furthermore, a birth defect is defined as “a physical or biochemical abnormality that is present at birth and that may be inherited or the result of environmental influence” [314]. By these definitions, *any chemical or mixture of chemicals* that result in MG developmental defects, especially those that result in *persistent changes in MG shape or function*, are *teratogens*.

This review has identified numerous examples of EDCs for which exposures during pregnancy are associated with alterations in maternal lactation and daughter’s timing of thelarche in humans [36, 91, 96, 99, 105] and cause similar effects in animal models [33, 35, 58, 118, 124, 125]. Since structural and functional alterations to the MG can clearly be driven by exposures during prenatal development, these exposures meet the definition of teratogenic. Furthermore, there is clear experimental support for the ability of EDCs that alter lactation, thelarche, and/or mammary density in humans to also alter MG development following prenatal exposure in rodents, such as BPA [117–119, 193, 194, 198, 227, 315] and PFOA [33, 58, 69, 124, 125]. In fact, in utero exposure to environmentally relevant doses of BPA in particular has explicitly been shown to induce detectable effects on MG development from embryonic stages that grow in magnitude through puberty and adult life and eventually manifest as mammary tumors [20]. It is thus likely that prenatal exposures to other chemicals that impair MG development and function in rodents, such as atrazine [30, 59, 60], may have similar teratogenic effects in humans if adequately studied.

## Conclusions

From population trends, epidemiological studies, and experimental evidence, it is clear that the breast is a vulnerable target of chemical exposures, and that exposures can produce multiple adverse effects in young women. Proper development of the MG is essential for reproduction and nutrition of mammalian species. We have documented *global* evidence for adverse environmental chemical influences on early breast development during puberty, shortened lactation duration in women, associations with increasing mammographic density, and breast cancer risk in premenopausal women. These effects are being missed in standard toxicology studies because of inadequate assessment of the MG. This is of special concern because effects on the MG often occur at lower doses than effects on other commonly measured outcomes such as vaginal opening or uterine weight. In addition, EDCs that induce MG effects have diverse modes of action and are not limited to estrogen receptor agonists, or even chemicals that affect the estrogen pathway. Thus, chemicals of concern for the breast are often being missed in standard toxicological testing. Mandatory collection and evaluation of mammary tissue on every chemical tested will inform epidemiological studies and enhance risk assessment of chemicals to prevent these serious outcomes.

We have provided an overview of key adverse outcomes for the MG that are caused by chemical exposures, focusing mostly on well-known EDCs with substantial evidence of adverse effects. Many more published studies, that we did not have space to include, describe effects of chemicals on the MG; more importantly, we emphasize that hundreds of EDCs and mixtures thereof have not yet been tested for effects on the MG. It is therefore likely that many more mammary toxicants and teratogens remain to be identified. Our recommendations to enhance methods for evaluating and classifying chemical effects on the MG will provide significant additional public health protection, particularly if they are applied globally in risk/hazard screening programs.

## Abbreviations

BBzP: Butyl benzyl phthalate
BPA: Bisphenol A
BPAF: Bisphenol AF
BPS: Bisphenol S
DBP: Dibutyl phthalate
DDE: Dichlorodiphenyldichloroethylene
DDT: Dichlorodiphenyltrichloroethane
DEHP: Di(2-ethylhexyl) phthalate
DEP: Diethyl phthalate
DES: Diethylstilbestrol
DiBP: Diisobutyl phthalate
DiDP: Diisodecyl phthalate
DiNP: Diisononyl phthalate
DMBA: 7,12-Dimethylbenz[a]anthracene
DMP: Dimethyl phthalate
EDC: Endocrine disrupting chemical
EOGRTS: Extended one-generation toxicity study
EPA: US Environmental Protection Agency
ER: Estrogen receptor
FDA: Food and Drug Administration
HRT: Hormone replacement therapy
ICH: The International Council for Harmonisation of Technical Requirements for Pharmaceuticals for Human Use
MBP: Monobutyl phthalate
MBzP: Monobenzyl phthalate
MCNP: Monocarboxy-isononyl phthalate
MCOP: Monocarboxyoctyl phthalate
MCPP: Mono(3-carboxypropyl) phthalate
MD: Mammographic density
MECPP: Mono(2-ethyl-5-carboxypentyl) phthalate
MEHHP: Mono(2-ethyl-5-hydroxyhexyl) phthalate
MEHP: Mono(2-ethylhexyl)
MEOHP: Mono(2-ethyl-5-oxohexyl) phthalate
MEP: Mono-ethyl phthalate
MG: Mammary gland
MMP: Mono-methyl phthalate
MNU: N-Methyl-N-nitrosourea
MOG: Modified one-generation
NOAEL: No observed adverse effect level
NTP: US National Toxicology Program
OECD: Organization for Economic Cooperation and Development
OCSPP: Office of Chemical Safety and Pollution Prevention
PAH: Polycyclic aromatic hydrocarbon
PBB: Polybrominated biphenyl
PBDE: Polybrominated diphenyl ether
PCB: Polychlorinated biphenyl
PCDD/F: Polychlorinated dibenzo-p-dioxins and furans
PFAS: Per- and polyfluorinated alkyl substances
PFDA: Perfluorodecanoic acid
PFHxS: Perfluorohexane sulfonic acid
PFNA: Perfluorononanoic acid
PFOA: Perfluorooctanoic acid
PFOS: Perfluorooctane sulfonic acid
PFOSA: Perfluorooctanesulfonamide
PM2.5: Particulate matter 2.5
PND: Postnatal day
RACB: Reproductive assessment by continuous breeding
TCDD: 2,3,7,8-Tetrachlorodibenzo-p-dioxin
TG: Test guideline
VO: Vaginal opening
WoS: Window of susceptibility

## Declarations

Silent Spring Institute is a scientific research organization dedicated to studying environmental factors in women’s health. The institute is a 501(c)3 public charity funded by federal grants and contracts, foundation grants, and private donations, including from breast cancer organizations.
